# Synchronous colorectal neoplasias: our experience about laparoscopic-TEM combined treatment

**DOI:** 10.1186/1477-7819-8-105

**Published:** 2010-11-25

**Authors:** Alessandro Spizzirri, Marco Coccetta, Roberto Cirocchi, Francesco La Mura, Vincenzo Napolitano, Maurizio Bravetti, Daniele Giuliani, Angelo De Sol, Eleonora Pressi, Stefano Trastulli, Micol Sole Di Patrizi, Nicola Avenia, Francesco Sciannameo

**Affiliations:** 1 General Surgery Department, St. Maria Hospital, Terni (TR), University of Perugia, Italy; 2 Head and Neck Surgery Department, St Maria Hospital, Terni (TR), University of Perugia, Italy

## Abstract

Synchronous colorectal neoplasias are defined as 2 or more primary tumors identified in the same patient and at the same time. The most voluminous synchronous cancer is called "first primitive" or "index" cancer. The aim of this work is to describe our experience of minimally invasive approach in patients with synchronous colorectal neoplasias.

Since January 2001 till December 2009, 557 patients underwent colectomy for colorectal cancer at the Department of General and Emergency Surgery of the University of Perugia; 128 were right colon cancers, 195 were left colon cancers while 234 patients were affected by rectal cancers. We performed 224 laparoscopic colectomies (112 right, 67 left colectomies and 45 anterior resections of rectum), 91 Transanal Endoscopic Microsurgical Excisions (TEM) and 53 Trans Anal Excisions (TAE). In the same observation period 6 patients, 4 males and 2 females, were diagnosed with synchronous colorectal neoplasias. Minimal invasive treatment of colorectal cancer offers the opportunity to treat two different neoplastic lesions at the same time, with a shorter post-operative hospitalization and minor complications. According to our experience, laparoscopy and TEM may ease the treatment of synchronous diseases with a lower morbidity rate.

## Introduction

Synchronous colorectal neoplasias, defined as 2 or more primary tumors identified in the same patient and at the same time, are caused by common genetic and environmental factors [1]. Since intraoperative palpation can miss up to 69% of the SN [2], currently, synchronous neoplastic lesions are usually diagnosed at a preoperative staging by colonoscopy or virtual colonoscopy; according to data from literature, 3% of the patients with SN are affected by different types of malignant lesions [3] while 33-55% show villous adenomas [4,5]. Literature also confirms the presence of primitive synchronous cancers [6]; malignant synchronous lesions are very rare, showing the following incidence: between 0,17% and 0,69% in case of 2-3 synchronous lesions, 0,19% in case of 4-5 synchronous lesions [7].

The most voluminous synchronous cancer is called "first primitive" or "index" cancer. When the index cancer is located in the caecum, the incidence of left colon synchronous cancers is higher than when the index cancer is located in the left colon [8,9].

Colorectal adenomas standard treatment is usually represented by endoscopic polypectomy; indeed only 5% of synchronous colorectal lesions require a surgical treatment [10].

When a villous adenoma or a T1 cancer is situated in the middle-lower rectum it is possible to perform a different surgical approach through a transanal endoscopic microsurgery (TEM) followed by a laparoscopic colectomy; this treatment can also be assessed in a reverse order. Firstly it is important to remove the obstructing lesion which can cause intestinal occlusion and later any other lesion, both malignant and benign ones. Multiple cancers patients show a worse prognosis compared to patients affected by single neoplastic lesions (5 year-survival rate incidence of 55%) [11].

The aim of this work is to describe our experience of minimally invasive approach in patients with synchronous colorectal neoplasias.

## Materials and methods

Since January 2001 till December 2009, 557 patients underwent colectomy for colorectal cancer at the Department of General and Emergency Surgery of the University of Perugia; 128 were right colon cancers, 195 were left colon cancers while 234 patients were affected by rectal cancers. We performed 224 laparoscopic colectomies (112 right, 67 left colectomies and 45 anterior resections of rectum), 91 Transanal Endoscopic Microsurgical Excisions (TEM) and 53 Trans Anal Excisions (TAE).

In the same observation period 6 patients, 4 males and 2 females, were diagnosed with synchronous colorectal neoplasias; 3 of them had no comorbility while the others were affected by hypertension (2 males and 1 female). Median age was 67.

All the patients had rectal tumors associated with a synchronous lesion with different locations and they all underwent a TEM through gas insufflation via a stereoscopic telescope associated with a laparoscopic colectomy for the synchronous colonic lesion:

- 1 patient showed a T1 rectal cancer associated with a voluminous sessile right colon adenoma;

- 3 patients were affected by voluminous rectal adenomas associated with colon cancers (2 right and 1 left colon cancers);

- 2 patients showed a rectal sessile adenoma associated with a voluminous villous right colon adenoma. (Table 1)

**Table 1:** Transmission electron microscopy results

PATIENTS	RECTUM		COLON	
	***ADENOMA***	***CARCINOMA***	***RIGHT***	***LEFT***

1		T1	Adenoma	

2	Adenoma		Carcinoma	

1	Adenoma			Carcinoma

2	Adenoma		Adenoma	

All patients underwent an endoscopic examination with biopsy. Virtual colonoscopy was required just in one case. A voluminous rectal sessile adenoma, whose size was larger than 4 cm, could not be removed by endoscopic excision. All patients underwent abdominal and chest CT scan. All the patients' rectal neoplasias were evaluated through transanal endoscopy and all of them were described as submucosal lesions. Three lesions were located on the posterior rectal wall, 2 on the anterior wall and 1 lesion was located on the left lateral rectal wall.

Surgical approach included a sequential exeresis characterized by the resection of the malignant lesion followed by the voluminous adenoma resection:

- One patient underwent TEM for the rectal lesion, followed by a laparoscopic right hemicolectomy for the voluminous villous adenoma.

- Two patients underwent a laparoscopic right hemicolectomy followed by TEM for a voluminous villous adenoma.

- The patient affected by left colon cancer associated with a voluminous villous rectal adenoma, underwent TEM followed by a laparoscopic left hemicolectomy. We decided to perform the endoscopic approach before the left hemicolectomy because of the large rectal tumor size, in order to ease the circular mechanical stapler transit. In this patient we decided to perform a left colectomy instead of an anterior resection of the rectum because the cancer was proximal and we avoided a larger resection.

- Two patients with rectal and right colon adenomas underwent TEM and a laparoscopic right hemicolectomy.

Each TEM procedure was performed using the full-thickness-excision technique considering the adenoma as a potential malignant lesion.

Median TEM operating time was 70 minutes, while laparoscopic resection required a mean time of 205 min. Intraoperative blood loss was modest for all patients. The most evident loss was approximately 300 cc in one of the patients undergoing a right hemicolectomy. Postoperative haemoglobin values were constant, ranging between 13,7 g/dl and 12,6 g/dl. We didn't register any complication either during the operative or postoperative time, but only one patient showed a modest hematochezia following a TEM procedure, which stopped spontaneously on the 3^rd ^postoperative day.

## Discussion

The high incidence of colorectal tumors leaded to the need for new surgical approaches. Till the Seventies, a preoperative diagnosis of synchronous colorectal cancers was quite rare (1.6%-4.3%), being mostly assessed by intraoperative bowel manipulation or accidentally [12-16].

In 1975 Heald e Bussey identified all the synchronous colorectal neoplastic lesions (3.5% out of 4884 cases) treated at St. Mark's Hospital in London from 1928 till 1970, showing that 31% of them had been accidentally discovered during intraoperative bowel manipulation while only 15% had already been diagnosed prior to operation (10% by clinical examination, 3% by barium enema, 2% by sigmoidoscopy) [12]. During the Seventies, the higher employment of preoperative examinations leaded to an increase of synchronous lesions diagnoses (7.6-8.1%) (Table 2) [17-20]. This issue was also stated by Fegiz in 1989 (1.6% of cases diagnosed by double contrast barium enema and 4,1% by colonoscopy) [21]. The frequency of synchronous neoplastic lesions is variable, ranging between 2.12% and 4.4% [22,23].

**Table 2:** * Preoperative examinations of lesions

PATIENTS	TEM	Laparoscopy
1	*Rectal cancer (I)*	*Right emicolectomy for adenoma(II)*

2	*Rectal adenoma (II)*	*Right emicolectomy for cancer(I)*

1	*Rectal adenoma (I)*	*Left emicolectomy for cancer (II)*

2	*Rectal adenoma (I)*	*Right emicolectomy for adenoma (II)*

* - (I) and (II): surgical sequence

A complete pre-operative study with colonoscopy or virtual colonoscopy is always necessary to perform a diagnostic evaluation of colon-rectum, allowing to detect the presence of synchronous lesions.

Colonoscopy is the most appropriate mean of investigation for colorectal cancer, but it cannot be performed in case of obstructive neoplastic lesions or in case of megacolon. In both cases either a double contrast barium enema, a virtual colonoscopy or an intraoperative colonoscopy [24,25] can be performed.

The recent employment of virtual colonoscopy has allowed an accurate study of the colonic segments upstream the stenosis [26]. Synchronous colon neoplastic lesions treatment is well known. Preoperative evaluation is very important especially when a laparoscopic approach is going to be performed, as the bowel cannot be palpated [27-29]. The treatment of synchronous colorectal neoplastic lesions can sometimes be complicated; for example, in case of neoplastic lesions in contiguous intestinal segments, such as the right and transverse colon or the left colon and rectum, it is necessary to perform an enlarged right hemicolectomy or an anterior resection of the rectum. In case of neoplastic lesions in distant colic segments, the employment of TEM reduces the probability of an anterior resection of the rectum.

The recent employment of TEM has reduced the need to perform an anterior resection of the rectum when tumors are located in two different and distant colonic segments. In case of right or transverse colon cancers associated with an extraperitoneal rectal cancer, a decrease of both laparoscopic and open total colectomy associated with a right anterior resection of the rectum has been registered [30,31].

In case of voluminous rectal adenomas or extra peritoneal T1 rectal cancers, a transanal local exeresis could be performed. In 1983 Buess performed TEM for neoplastic lesions whose diameter was smaller than 25% of the entire bowel circumference and without lymphonodal infiltration [32-34].

In literature, a sequential employment of TEM and laparoscopic resection of synchronous colorectal malignant lesions is described by Ikeda [35]. This mini-invasive approach allows a significant rectum sparing.

## Results and conclusions

Our experience showed that the combination of TEM and laparoscopic hemicolectomy was characterized by an uncomplicated post-operative time. The current disease free survival is 83% (1/6) as one of the patients with right colon carcinoma was diagnosed with a singular liver metastasis 3 years after the first surgical approach. We also registered no complication compared to the standard transanal resection with a significant reduction of both the hospital stay and the complication rates compared to conventional anterior resection of rectum.

The final histological diagnoses were low grade dysplasia adenomas of the rectum while only one of the rectal lesions was classified as a T1 cancer. The right colon lesions were classified as high grade dysplasia adenomas, one of which had some focus of adenocarcinoma, while the other 2 patients with right colon tumors were classified as B1 and B2 stages according to Dukes' classification. The patient with the left colon tumour was classified as a B1 stage. (Table 3)

**Table 3:** Definitive histological diagnoses

PATIENTS	RECTUM		COLON	
	***ADENOMA***	***CARCINOMA***	***RIGHT***	***LEFT***

1		T1	High grade dysplasia	

2	1) Low grade dysplasia 2) Low grade dysplasia		1) B1 (carcinoma) 2) B2 (carcinoma)	

1	Low grade dysplasia			B1 (carcinoma)

2	1)Low grade dysplasia 2) Low grade dysplasia		1) High grade dysplasia 2) High grade dysplasia with focus of adenocarcinoma	

All the resection margins were disease free.

Minimal invasive treatment of colorectal cancer offers the opportunity to treat two different neoplastic lesions at the same time, with a shorter post-operative hospitalization and minor complications. According to our experience, laparoscopy and TEM may ease the treatment of synchronous diseases with a lower morbidity rate. Our aim is to increase the observed number of cases in order to minimize the complications and obtain better results.

## Competing interests

The authors declare that they have no competing interests.

## Authors' contributions

AS drafted the article, updated the references and checked the numbers and percentages. MC drafted the article, updated the references and checked the numbers and percentages. RC drafted the article, updated the references and checked the numbers and percentages. FL cooperated in writing the article and translated it into English. VN made the tables. MB searched for the references and formatted the article. DG searched for the references and formatted the article. AD collected patients' data. EP chose the most useful and interesting articles in literature about the field. ST searched for the references and collected the patients' consent. MD searched for the references and collected the patients' consent. NA supervised the article production. FS allowed the collection of the patients' data and supervised the whole work making. All authors read and approved the final manuscript
